# Expression of caveolin-1 in tooth germ, ameloblastoma and ameloblastic carcinoma

**DOI:** 10.4317/medoral.24151

**Published:** 2020-10-09

**Authors:** Celeste Sánchez-Romero, Vanesa Pereira-Prado, Estefanía Sicco, Mariana Suarez, Gabriel Tapia-Repetto, Ramón Carreón-Burciaga, Rogelio Gónzalez-Gónzalez, Mariana Villarroel-Dorrego, Marco Meleti, Nelly Molina-Frechero, Ronell Bologna-Molina

**Affiliations:** 1 PhD. Molecular Pathology Area, Faculty of Dentistry, University of the Republic, Uruguay; 2 MSc. Molecular Pathology Area, Faculty of Dentistry, University of the Republic, Uruguay; 3 PhD. Oral Pathology, school of Dentistry, Universidad Juarez del Estado de Durango, Mexico; 4 PhD. Oral Histopathology Laboratory, Universidad Central de Venezuela, Venezuela; 5 PhD. Department of Medicine and Surgery, Oral Medicine and Laser Surgery Unit, Centro Universitario di Odontoiatria, University of Parma, Italy; 6 PhD. Health Care Department, Universidad Autónoma Metropolitana, Xochimilco, Mexico

## Abstract

**Background:**

The caveolin-1 protein (structural component of membrane caveolae) plays important roles in several biological functions, such as endocytosis, cell adhesion, and cell signaling. However, this protein has been associated with mechanisms of tumorigenesis in several neoplasms. The expression patterns and roles of caveolin-1 in the oral epithelium and in embryonic and odontogenic tumor tissues are still unclear.

**Material and Methods:**

The expression of caveolin-1 was evaluated in samples of the normal gingival epithelium (n=7), human tooth germ (TG) (n=12), ameloblastoma (AM) (n=83), and ameloblastic carcinoma (AC) (n=9) by immunohistochemistry. Additionally, AM samples were analyzed by qRT-PCR and Western blot.

**Results:**

Most TG (91.7%), AM (73.5%) and AC (100%) samples showed diverse patterns of immunohistochemical positivity for caveolin-1, while only one gingival sample was positive. The transcript levels of cav-1 were significantly upregulated by 14.9-fold in AM tissue (*P* = 0.0014) compared to those in normal gingival epithelial tissue, as shown by qRT-PCR. Presence of caveolin-1 protein was confirmed by Western blot analysis. The caveolin-1 immunoexpression patterns throughout the stages of TG show its importance during odontogenesis.

**Conclusions:**

The overexpression of caveolin-1 in AM and AC compared to its expression in normal gingival epithelium (adult tissue) suggests a possible role of caveolin-1 in protumoral events, but due to the similar immunoexpression observed in AM and AC, caveolin-1 may not necessarily participate in the malignant transformation process. However, future studies are needed to clarify and confirm these hypotheses.

** Key words:**Ameloblastoma, ameloblastic carcinoma, caveolin-1, immunohistochemistry, real-time polymerase chain reaction.

## Introduction

The protein caveolin-1 is a 21–24 kDa scaffold protein, and in addition to caveolin-2 and -3, caveolin-1 represents the main structural component of the caveolae or flask-shaped invaginations of the plasma membrane and plays important roles in several biological functions, such as endocytosis, cell adhesion, and cell signaling; therefore, caveolin-1 is widely expressed in most cell types of normal human tissues (1,2). In the context of cancer, caveolin-1 is the isoform that is mainly associated with tumoral mechanisms, such as malignant transformation, angiogenesis, tumor stage, metastasis, chemotherapeutic response, and prognosis. For some tumors, caveolin-1 overexpression is associated with protumoral mechanisms; in contrast, for other cancer types, the loss of caveolin-1 expression is associated with poor prognosis. The prognostic value of this marker is strongly cancer-specific (3).

There are few studies describing caveolin-1 expression in tumors arising intraorally or within the gnathic bones, such as primordial odontogenic tumors, malignant salivary gland tumors, oral squamous cell carcinoma and potentially malignant oral lesions (4–7). Moreover, there is a report of caveolin-1 immunopositivity in odontogenic cysts and ameloblastoma (AM), which is the most common benign epithelial odontogenic tumor that is characterized by its potential for local invasion and tendency of recurrence; hence, most patients are treated with radical surgery (3,8). However, to the best of our knowledge, this is the first study whose objective was to evaluate caveolin-1 expression by immunohistochemistry in a considerable number of AMs (also analyzing the cav-1 gene transcript expression by qRT-PCR) and in human tooth germ (TG) and ameloblastic carcinoma (AC), which is the malignant counterpart of AM; although it is rare, AC is the most frequent malignant odontogenic tumor and presents higher recurrence rates and metastatic potential than other malignant odontogenic tumors (9).

## Material and Methods

- Case selection

Formalin-fixed, paraffin-embedded tissue samples of 7 normal gingival tissues, 12 human TG (3 in bud stage, 3 in cap stage, and 6 in bell stage of development), 83 AM (57 solid/conventional tumors and 26 unicystic tumors), and 9 AC were retrieved from the files of the Molecular Pathology and Histology areas at the School of Dentistry of Universidad de la República (Uruguay), the Laboratory of Oral Pathology at the Dental School of Piracicaba, University of Campinas (Brazil), and the Department of Pathology of Integra Cancer Center (Guatemala).

The specimens were harvested from nondecalcified portions of the tumors. For the immunohistochemical analyses, 2-μm sections were treated with a heat retrieval solution (Reveal Decloaker, RTU; Biocare Medical, Pacheco, CA) to expose the antigenic epitopes. The endogenous peroxidases were blocked with 0.9% hydrogen peroxide for 5 min. The tissue sections were incubated with a primary antibody against Caveolin-1 (polyclonal; 1:100 dilution, Santa Cruz Biotechnology, Dallas, TX) for 60 min and then incubated with a biotinylated antimouse/antirabbit antibody and a streptavidin–horseradish peroxidase complex for 40 min each (Mouse/Rabbit ImmunoDetector Biotin Link & HRP Label; Bio SB, Santa Barbara, CA). For the negative control sections, the primary antibody was omitted, and human lung tissue was used as a positive control. The reaction products were visualized using the 3,3’-diaminobenzidine–H2O2 substrate (Biocare Medical), and the sections were counterstained with Mayer’s hematoxylin.

For cytoplasmic and/or membranous positivity in odontogenic epithelial cells, quantification was performed visually using an optical microscope (Eclipse Ci-L, Nikon, Japan) within the whole tissue section according to the following semi-quantitative scale: a score of 0 (“essentially no staining”) was established for negative immunohistochemical staining or positive immunohistochemical staining of < 5% of the cells; a score of + (“weak-moderate”) indicated staining of 5 to 50% of the cells, and a score of ++ (“strong positive”) indicated staining of >50% of the cells. Positive staining in the nucleus and in the mesenchymal/stromal components was recorded as “present” or “absent”. The results were analyzed descriptively. The Mann–Whitney U-test was used to assess the differences in caveolin-1 expression within AM subtypes.

- Extraction of total RNA and reverse transcription

For the molecular biology assay, 2 AM human samples were used. As a control, 1 normal gingival tissue sample from a patient who underwent surgery at the School of Dentistry of Universidad de la República (Uruguay) was used (10). All the samples were fixed in formalin and embedded in paraffin.

The total RNA was extracted using the Quick-RNA FFPE Kit (Zymo Research, USA) according to the manufacturer’s instructions. The integrity of the total RNA was evaluated by GoodView (SBS Genetech, China) staining in an agarose gel. Reverse transcription was performed with 5 µg of the total RNA using the SuperScript® III Frist-Strand Synthesis system for RT-PCR (Life Technologies, USA) with oligo(dT) primers according to the manufacturer’s instructions. The reaction mixture (20 µL) was incubated at 65°C for 5 min, 50°C for 50 min, 85°C for 5 min, and then 37°C for 20 min. The RNA and cDNA obtained were quantified using a NanoDrop DS-11 spectrophotometer (Denovix, USA).

- Primer design and real-time PCR

The primers were developed using the web application Primer-BLAST NCBI22, and their specificity was evaluated against the human genome using the Basic Local Alignment Search Tool. The primer sequences were as follows: gapdh (normalizing gene)-specific primers (forward 5′-CAC CAT CTT CCA GGA GCG AG-3′ and reverse 5′-GAC TCC ACG ACG TAC TCA GC-3′) and cav-1-specific primers (forward 5′-CAG TGC ATC AGC CGT GTC TA-3′ and reverse 5′-TCT GCA AGT TGA TGC GGA CA-3′).

q-PCR was performed using the DTlite Real-Time PCR Thermal Cycler System (DNA Technology, Russia) and was carried out using Brilliant® SYBR® Green q-PCR (Bioline Meridian Life Science, USA). The amplification was performed in duplicate with systematic negative controls (nontemplate control containing no cDNA). The q-PCR protocols included an initial denaturation step at 95°C for 2 min, followed by 40 cycles: 5 s at 95°C, 20 s at 62°C annealing temperature, and 15 s at 90°C. The amplification phase was followed by a ramp of 90°C to 40°C at 0.5°C/s, and the data were collected in continuum to obtain a single product dissociation curve.

The variations in the transcription levels (normalized by reference genes) were analyzed using the 2-∆∆CT method (11). For statistical significance, a nonparametric t test was used (GraphPad Software v.5.01). The significance level was set to 0.05. The data are presented as the mean ± SD of biological replicates.

- Western Blot

In two cases of AM, protein extraction was performed using a Qproteome FFPE tissue kit (Catalog Number 37623, QIAGEN, Hilden, Germany), following the manufacturer’s recommendations. The protein concentration was determined using the Bradford method with a spectrophotometer DeNovix DS-11. From each extraction, only 10 to 20 μg of protein were used and separated on 12% PAGE/SDS (polyacrylamide gel electrophoresis/sodium dodecyl sulfate), 100 V for 30 minutes. Electrotransference to polyvinylidene difluoride membranes over 2 hours at ambient temperature was performed (Hoefer Blot Module). Blockage was performed using buffer TNE (10 mM Tris-HCl, pH 7.5; 2.5 mM EDTA, pH 8.0, and 50 mM NaCl), 1% Tween-20, and ambient temperature for 1 hour. Three washings were performed for 5 minutes each with TBST buffer. Incubation of the primary antibodies anti-caveolin-1 (1:1100), and anti-α-actin (1:500) with the membrane was performed at 4°C for 2 hours under soft stirring. Then, the secondary antibody (goat-anti-mouse-HRP conjugated secondary antibody) was added at room temperature (diluted 1/5000) for 1 hour under soft stirring. Detection was performed using an Opti-4CN substrate kit (Catalog Number 970-3210, BIORAD, Hercules, CA).

## Results

**Figure 1:**
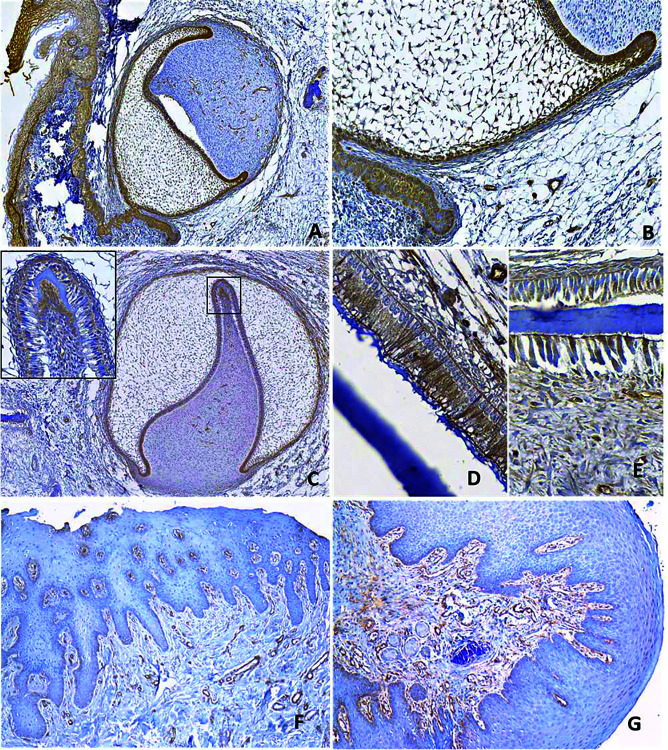
Caveolin-1 immunostaining in non-tumoral tissues: human tooth germ (A-E) and gingiva (F-G). In the early stages of development (late cap and permanent bud) (A, B), only the epithelial elements of the enamel organ exhibited strong expression; in the ectomesenchyme, only the blood vessels were immunostained. In the late developmental stages (C-E), in addition to the epithelial elements, the secretory odontoblasts and the adjacent dental papilla were positive, while the presecretory ameloblasts showed weak expression (C, inset). The secretory ameloblasts (D), the odontoblasts, and the dental papilla (E) exhibited strong expression of caveolin-1 in the late bell stage. In two samples of normal epithelial gingival tissue (F,G) scarce immunohistochemical staining was observed, mainly restricted to the basal layer. (Immunohistochemistry, original magnification: A,F,G: 100x, B: 200x, C: 50x, D, E, inset (C): 400x).

Of the 12 TGs, only one sample was negative for caveolin-1, and most samples presented strong positivity (++) in the epithelial components. Immunostaining was also observed in the mesenchymal components of the bell stage, such as the secretory odontoblasts and the adjacent dental papilla, while in the early stages (bud and cap), the positivity was limited to the epithelial components (stellated reticulum, inner and outer enamel epithelium). Most of the blood vessels and adjacent osteoblasts were positive (Fig. 1).

**Figure 2:**
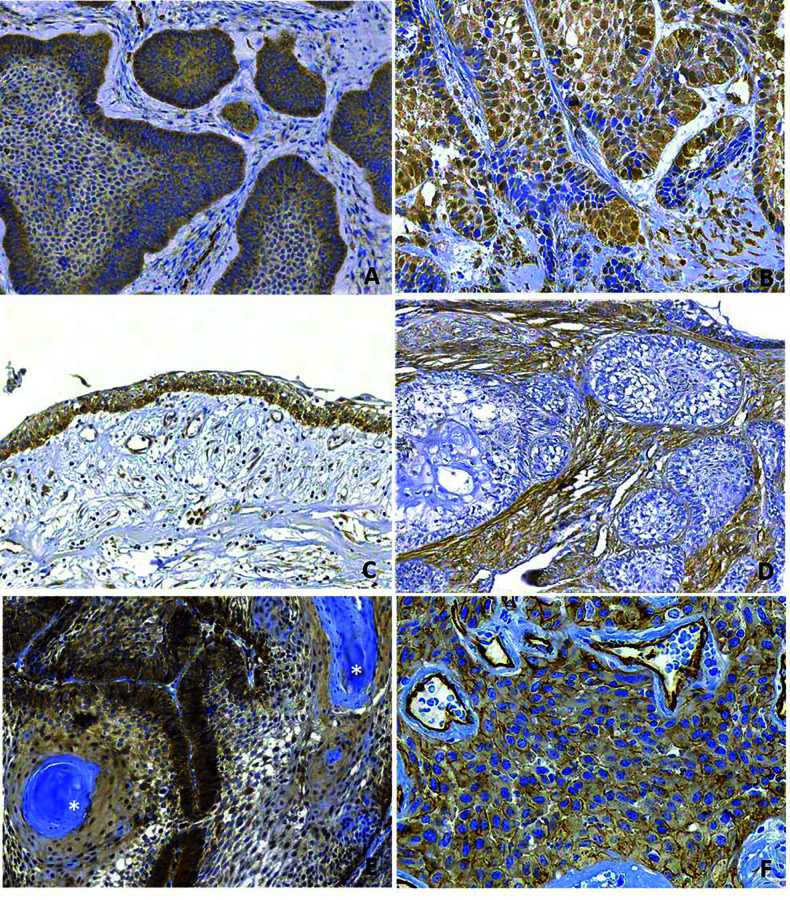
Patterns of caveolin-1 expression in ameloblastoma (A-D) and ameloblastic carcinoma (E,F). Cytoplasmic/membranous staining (A) and cytoplasmic/membranous with nuclear positivity (B) were the most common staining patterns in the solid (A, B) and unicystic (C) ameloblastomas. One ameloblastoma sample showed strong positivity in the stromal fibroblasts and negative tumor islands (F). In ameloblastic carcinomas, a strong and diffuse cytoplasmic immunostaining of caveolin-1, with occasional nuclear positivity, and negativity of the keratinization areas (asterisk) were observed (E). In some areas, a strong membranous staining pattern was clearly seen (D). (Immunohistochemistry, original magnification: A,D,E: 200x, B, F: 400x C:100x).

Immunoexpression of caveolin-1 in the cytoplasm and membranes of the epithelial tumor cells of the AM samples was negative in 22 (26.5%) samples and positive in 61 (73.5%) samples (Fig. 2). Most of the negative samples were solid ameloblastomas (SA) (17, 29.8%), and most of the positive samples were scored as + “weak-moderate staining” (42.2% of all the AM samples); there was a similar distribution in both SA and unicystic ameloblastomas (UA) (Mann–Whitney U-test, *p*=0,834), representing 28.9% and 23.1% of each subtype, respectively.

Of the total AM samples, 26 (31.3%), including 17 (29.8%) of SA and 7 (26.9%) of UA, were given a score of “strong positive”. Cytoplasmic/membranous staining was the most common staining pattern in both SA and UA (Fig. 2). Several AM samples (n=13,15.6%) showed focal nuclear positivity (Fig. 2) (Table 1). The occasional and rare stromal inflammatory cells and fibroblasts showed caveolin-1 positivity; however, one SA sample showed strong and diffuse staining of the stromal fibroblasts (Fig. 2). Other positive cell types in the samples included the endothelium and smooth muscle of blood vessels (which served as an internal control of the reaction), osteocytes, perineural and adipose tissue.

**Table 1:**
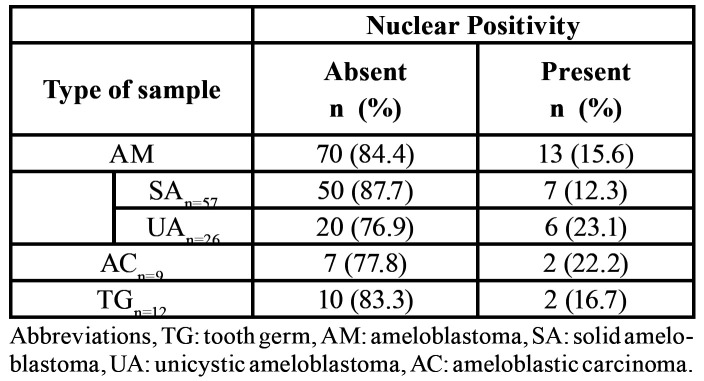
Nuclear immunoexpression of caveolin-1 in each studied entity.

All 9 AC samples were positive for caveolin-1 and exhibited similar distributions within the categories of “weak-moderate” (n=5, 55.6%) and “strong positive” (n=4, 44.4%). Two samples (22.2%) presented positive staining in the nucleus. Focal negative areas were observed in the central areas in the keratinization or necrosis processes (Fig. 2).

**Figure 3:**
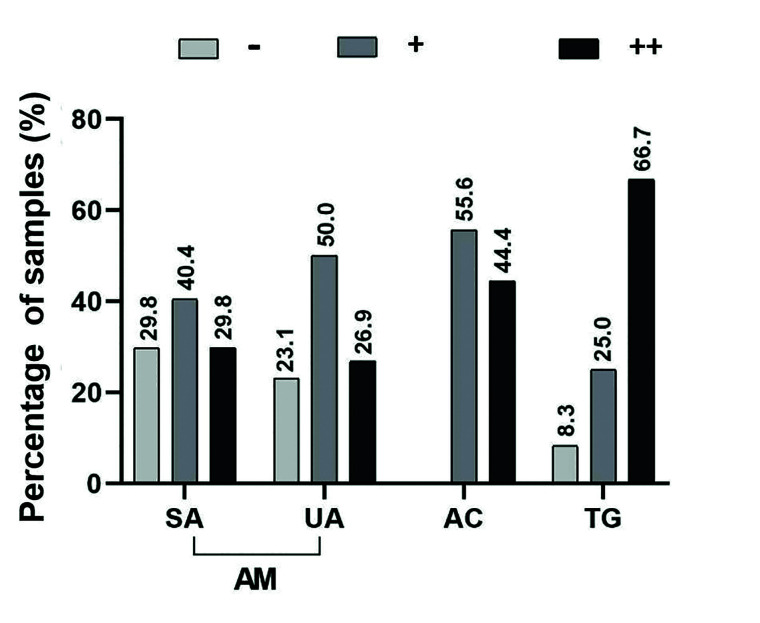
Immunoexpression of caveolin-1 in each studied entity.

The immunoexpression of caveolin-1 in each studied entity is summarized in Table 2 and Fig. 3.

The normal epithelial gingival tissue samples were predominantly negative for caveolin-1 immunostaining (Fig. 1) (six were negative, and one exclusively showed nuclear staining in the spinous layer), according to the RT-PCR control results shown below.

**Figure 4:**
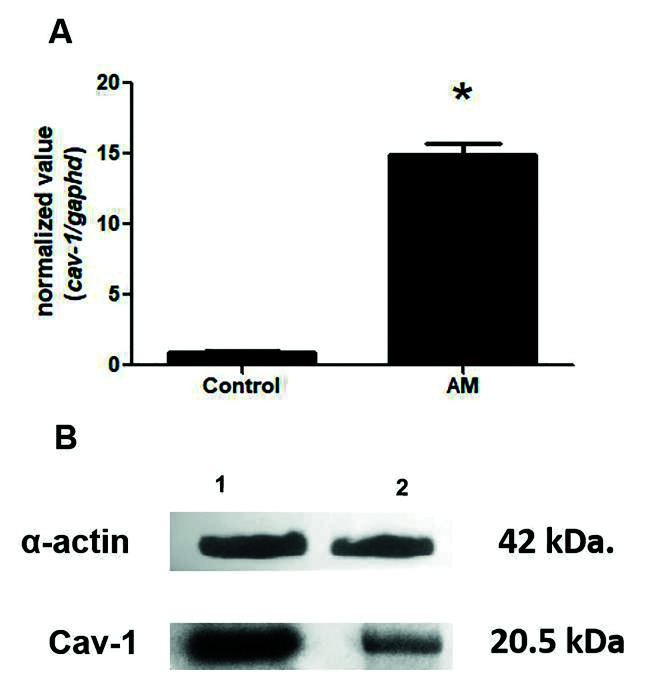
Expression of the cav-1 gene transcript in AM (A) and protein expression by Western blot analysis (B). A: qRT-PCR analysis of cav-1 transcript expression was performed on the AM and control samples. Two independent AM samples and one normal control sample were examined in duplicate. The data are means ± SDs of biological replicates. Asterisks indicate significant differences (* *P* < 0.05) between the AM and normal control samples. B: Western blot analysis of caveolin-1 (20.5 kDa) protein expression and α-actin (positive control) in two samples of ameloblastomas.

Qualitative RT-PCR was used to measure the differential expression of the cav-1 gene in the AM samples and in the normal controls. As shown in Fig. 4, the transcript levels of cav-1 (*P* = 0.0014) were upregulated by 14.9-fold in AM. Finally, the presence of caveolin-1 protein was confirmed by Western blot analysis in both of the AM samples studied (Fig. 4).

**Table 2:**
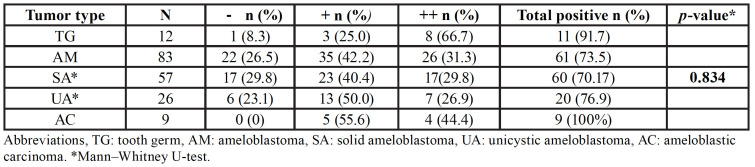
Immunoexpression of caveolin-1 in each studied entity.

## Discussion

Since caveolin-1 is needed for caveolae formation, it is expressed in most cell types and has a wide variability of functions and molecular interactions, mainly between integrin receptors and intracellular signaling molecules, for which the microdomains (one transmembrane and two cytoplasmic) of the caveolae are crucial (2). Additionally, caveolin-1 is associated with endocytosis, extracellular matrix organization, cholesterol distribution, cell migration and signaling (12). Failure of caveolae formation due to the altered expression of the caveolin genes is associated with a wide variety of diseases, such as cardiovascular disease, lipodystrophy, muscular dystrophy and some types of cancer (13). Several signal transduction molecules, such as epidermal growth factor (EGF) receptor, H-RAS, Src family tyrosine kinase, and protein kinase C, are regulated by caveolin-1 (1).

Among the physiological and developmental roles of caveolin-1, its expression during mouse odontogenesis, particularly in the lingual portion of the TG in the initial stages of odontogenesis, has been reported. Moreover, with the progression of the developmental stages, a gradual increase in caveolin-1 in the inner enamel epithelium, cervical loop, and ameloblasts was observed, which was confirmed by real-time polymerase chain reaction. This observation suggests a role for caveolin-1 in mouse tooth development, especially in the differentiation and organization of odontogenic tissues. Additionally, considering the relationship between caveolae/caveolin-1 and calcium transport mechanisms, the authors considered that caveolin-1 must participate in the passage of calcium to the ameloblasts (14). The immunohistochemical results of that study are similar to the result of our study; however, the authors did not detect caveolin-1 expression in the odontoblasts and the adjacent dental papilla. Therefore, we suggest that the same mechanisms could be related to the differentiation of odontoblasts and to the production of dentin matrix. Similar distributions and correlations with cytodifferentiation and mineralization processes have been attributed to the protein connexin-43 in human tooth germ (15,16).

In general, caveolin-1 seems to be important for carcinogenesis, since it is overexpressed or mutated in several types of human solid cancers (6). However, the association of caveolin-1 with protumoral or antitumoral mechanisms depends on the cancer subtype and organ of origin (17,18). For example, a tumor-promoting role of caveolin-1 has been found in renal and prostate cancers and in lung and bladder squamous cell carcinomas (SCCs). On the other hand, in cutaneous SCC, esophagus, and lung adenocarcinomas, caveolin-1 seems to play an inhibitory role. The role of caveolin-1 in other tumors, such as breast, pancreas, and thyroid cancer and cervical and head and neck SCC, is still controversial (18). Other authors have suggested that the dual role of caveolin-1 is based on tumor progression; caveolin-1 may induce cell cycle arrest and apoptosis in the early stages (tumor suppression), whereas an increase in the expression of caveolin-1 may promote tumor invasion, angiogenesis, and metastasis in later stages (19). In summary, the specific roles of caveolin-1 in tumorigenesis remain controversial for many tumors (17).

In oral tumors, caveolin-1 has been studied mainly in oral squamous cell carcinoma (OSCC) and its carcinogenesis and tumor progression processes, and caveolin-1 exhibits increased immunoexpression in OSCC compared to normal mucosa, dysplastic lesions and oral lichen planus (5,6). However, downregulation of caveolin-1 has been observed in metastatic OSCC when compared with primary tumors. Additionally, reduced caveolin-1 expression or its inactivation by mutation has been suggested to play a role in the pathogenesis of this malignancy, indicating a biphasic role in oral carcinogenesis (6,20). It has been suggested that caveolin-1 may activate metastasis and invasive capacities of OSCC cells and could be used as a prognostic marker for OSCC, since its high expression at metastatic lymph nodes, and worse outcome, have been observed (21). Therefore, OSCC joins the list of tumors for which the role of caveolin is controversial, and there are several theories about its possible roles in the pathogenesis and progression of OSCC (19).

Studies of salivary gland tumors found that caveolin-1 expression was inversely correlated with the duration of the tumor, clinical stage, histologic grade, and microvascular density in mucoepidermoid carcinoma and with the proliferation index in pleomorphic adenomas. In other studies, caveolin-1 expression was not correlated with the tumor size and stage in benign and malignant salivary gland tumors, suggesting that this protein may function as a tumor suppressor in these neoplasms; however, its clinical/prognostic implications are not yet clear (4,22).

There are only two immunohistochemical studies reporting the expression of caveolin-1 in odontogenic tumors and cysts. One study reported variable caveolin-1 positivity in the epithelial lining of the primordial odontogenic tumor, associating its expression with different stages of cellular differentiation and tumoral transformation (7). Another study found that many types of odontogenic cysts showed higher expression (in 100% of the 41 cases) than AM (in 58% of 34 cases). It was suggested that the aggressiveness of AM could be enhanced by the loss of caveolin-1 (3). In our study, 73.5% of the 84 AM samples were immunohistochemically positive for caveolin-1, and this overexpression was confirmed by the significantly increased expression of the cav-1 gene transcript in AM compared to the normal tissue, as determined by RT-PCR. Additionally, the presence of caveolin-1 protein was confirmed by Western blot in AM samples. The immunoexpression of caveolin-1 exhibited a similar distribution in SA and UA, showing a globally higher positivity rate and suggesting that in addition to this protein being involved in AM pathogenesis, it is not associated with the more aggressive behavior of SA compared to UA. In our study, we evaluated caveolin-1 immunoexpression in AC for the first time. All 9 samples (100%) were positive with a staining pattern similar to that of AM; however, the proportion of negative/positive samples is difficult to estimate due to the small AC sample size, since it is a rare tumor. Because there were no significant differences in caveolin-1 immunoexpression between AM and AC, this protein may not be involved in the malignant transformation process.

We observed that occasional and focal stromal inflammatory cells and fibroblasts showed caveolin-1 positivity in most AM samples. Strikingly, one SA sample showed strong and diffuse staining of the fibroblasts of the tumoral stroma and predominantly negative staining of the tumor cells; this staining pattern resembled the caveolin-1 immunostaining pattern of cancer-associated fibroblasts (CAFs) in the stroma of some malignant tumors, such as breast, colorectal and kidney cancer and metastatic melanomas. Caveolin-1-positive CAFs are able to remodel tumor microenvironments *in vivo*, facilitating tumor invasion and increasing metastatic potential, which correlates with low survival in breast cancer and with other clinicopathologic variables, such as pleural invasion, shorter recurrence and predominantly solid subtypes in lung cancer (23). Abundant myofibroblasts (a-SMA positive) with a histological appearance similar to that of the sample mentioned above were observed in more than 50% of the AM samples in a previous study, which associated the abundant presence of myofibroblasts and expression of MMP-2 with a more aggressive infiltrative behavior (24). Additionally, in a cell culture study, ameloblastoma-associated fibroblasts (AAFs) tended to stimulate proliferation and induce invasion more than gingival fibroblasts (25). The presence of CAFs has not been studied in AC; however, we did not observe an expression pattern of caveolin-1-positive CAFs in our AC samples.

Metabolic alterations are crucial for tumor cell survival, and in recent studies, caveolin-1 has been found to modulate cell metabolism with a focus on glycolysis (via hypoxia inducible factor 1α), mitochondrial bioenergetics, glutaminolysis, fatty acid metabolism, and autophagy in cancer cells (12). The overexpression of proteins associated with glucose metabolism (Glut-1), hypoxia (HIF-1a) and fatty acid synthesis (FASN) has been described in AM and AC (8,26). This finding suggests that caveolin-1 may play roles in the regulation and/or alteration of the metabolic pathways in these tumors.

Several authors have suggested that when caveolin-1 is located in the nucleus, its role is related to tumor suppression and gene regulation (27–29). This role could be related to the activation of VEGF, which induces caveolin-1 translocation from the caveolae to the nucleus in endothelial cells, transforming bioactive molecules into transcription factors (28).

Caveolin-1 localization in the nuclei of ovarian cancer cells has been reported, suggesting that soluble caveolin-1 is transported into the nucleus to regulate gene expression; however, no pathway has been identified, although caveolin-1 downregulates the expression of cyclin D1, which functions in the control of genes related to proliferation (29).

The main limitations of our study are the lack of correlation with clinical variables or with specific cell signaling pathway biomarkers and the limited number of AC samples due to their scarcity.

## Conclusions

The caveolin-1 immunoexpression patterns throughout the stages of TG show its importance during odontogenesis. The similar patterns of caveolin-1 overexpression in AM and AC suggest that it could play a role in protumoral events, probably through metabolic alterations, but not necessarily participate in the malignant transformation process. However, to confirm these hypotheses and because caveolin-1 is involved in a wide range of protumoral and antitumoral mechanisms, in future studies, the expression of caveolin-1 should be associated with clinical variables or with proteins involved in signaling pathways, angiogenesis, hypoxia and metabolic alterations.
